# Biomass pretreatment affects *Ustilago maydis *in producing itaconic acid

**DOI:** 10.1186/1475-2859-11-43

**Published:** 2012-04-05

**Authors:** Tobias Klement, Sofia Milker, Gernot Jäger, Philipp M Grande, Pablo Domínguez de María, Jochen Büchs

**Affiliations:** 1 AVT - Biochemical Engineering, RWTH Aachen University, Worringerweg 1, D-52074 Aachen, Germany; 2 Institut für Technische und Makromolekulare Chemie (ITMC), RWTH Aachen University, Worringerweg 1, D-52074 Aachen, Germany

**Keywords:** *Ustilago maydis*, Itaconic acid, Lignocellulose, Pretreatment, Seawater, RAMOS

## Abstract

**Background:**

In the last years, the biotechnological production of platform chemicals for fuel components has become a major focus of interest. Although ligno-cellulosic material is considered as suitable feedstock, the almost inevitable pretreatment of this recalcitrant material may interfere with the subsequent fermentation steps. In this study, the fungus *Ustilago maydis *was used to produce itaconic acid as platform chemical for the synthesis of potential biofuels such as 3-methyltetrahydrofuran. No studies, however, have investigated how pretreatment of ligno-cellulosic biomass precisely influences the subsequent fermentation by *U. maydis*. Thus, this current study aims to first characterize *U. maydis *in shake flasks and then to evaluate the influence of three exemplary pretreatment methods on the cultivation and itaconic acid production of this fungus. Cellulose enzymatically hydrolysed in seawater and salt-assisted organic-acid catalysed cellulose were investigated as substrates. Lastly, hydrolysed hemicellulose from fractionated beech wood was applied as substrate.

**Results:**

*U. maydis *was characterized on shake flask level regarding its itaconic acid production on glucose. Nitrogen limitation was shown to be a crucial condition for the production of itaconic acid. For itaconic acid concentrations above 25 g/L, a significant product inhibition was observed. Performing experiments that simulated influences of possible pretreatment methods, *U. maydis *was only slightly affected by high osmolarities up to 3.5 osmol/L as well as of 0.1 M oxalic acid. The production of itaconic acid was achieved on pretreated cellulose in seawater and on the hydrolysed hemicellulosic fraction of pretreated beech wood.

**Conclusion:**

The fungus *U. maydis *is a promising producer of itaconic acid, since it grows as single cells (yeast-like) in submerged cultivations and it is extremely robust in high osmotic media and real seawater. Moreover, *U. maydis *can grow on the hemicellulosic fraction of pretreated beech wood. Thereby, this fungus combines important advantages of yeasts and filamentous fungi. Nevertheless, the biomass pretreatment does indeed affect the subsequent itaconic acid production. Although *U. maydis *is insusceptible to most possible impurities from pretreatment, high amounts of salts or residues of organic acids can slow microbial growth and decrease the production. Consequently, the pretreatment step needs to fit the prerequisites defined by the actual microorganisms applied for fermentation.

## Background

Since fossil fuels are limited, many current research projects are investigating the utilization of renewable resources to ensure the sustainable production of biofuels and platform chemicals. Recently, most of these approaches have focused on producing alcohols from starch which competes with the food supply chain. Moreover, these approaches waste most of the plant biomass. Thus, new research is focusing on utilizing ligno-cellulose as the prime raw material for biofuel production [1] and constructing new biocatalysts for this purpose [2].

Itaconic acid (C_5_H_6_O_4_, methylene succinic acid) is an unsaturated dicarbonic acid with pKs values of 3.84 and 5.55 and a molecular weight of 130.1 g/mol. Due to its interesting chemical attributes, several studies have declared itaconic acid to be one of most promising platform chemicals derived from biomass [3-5]. Analogous to other organic acids such as citric acid or lactic acid, itaconic acid is mainly supplied by biotechnological processes with the fungus *Aspergillus terreus *(*A. terreus*) [6,7]. So far, this acid is mostly applied in the polymer industry for producing nitrilon, in the ion exchange chromatography sector, papermaking, and waste water treatment [7]. For synthesizing promising biofuel components, new catalytic conversions from itaconic acid to products such as 3-methyltetrahydrofuran (3-MTHF) or 2-methylbutanediol (2-MBDO) have been realized [8]. Detailed reviews regarding itaconic acid production, its biosynthesis, and its economic development can be found in Wilke and Vorlop [6] and Okabe et al. [7].

In this current study, itaconic acid fermentation was carried out with the mould fungus *Ustilago maydis *(*U. maydis*), an important fungal model organism in many different research fields, such as plant-pathogen interaction, mating, and signal transduction [9]. From an industrial point of view, *U. maydis *can also produce several secondary metabolites such as glycolipids, iron-chelating siderophores, and tryptophan metabolites [10]. In 1955, the production of itaconic acid was firstly described for *U. maydis *[11]. In addition, in their study, Guevarra and Tabuchi [12] proposed the production of several organic acids with this fungus, among others, itaconic acid. In contrast to *A. terreus*, the main advantage of *U*. *maydis *is its ability to grow in a yeast-like morphology as single cells in its haploid form. Therefore, several severe problems with filamentous fungi such as elevated viscosity, hindered oxygen transfer, sensitivity to hydromechanical stress, and laborious handling of spores are avoided.

In recent studies, different pretreatment methods have been investigated that facilitate the fractionation of lignocellulose [13] as well as the hydrolysis of cellulose and/or hemi-cellulose [14]. This fractionation yields xylose from hydrolysed hemicellulose, whereas the remaining cellulosic fraction is hydrolysed to glucose after the pretreatment. Both xylose and glucose can be converted to the platform chemical itaconic acid in a fermentation process [15]. Nevertheless, pretreatment may heavily influence the subsequent fermentation process or even completely inhibit the growth of *U. maydis*.

The aim of this current study is to characterize *U. maydis *on a small scale level in shake flasks and to evaluate its potential regarding the production of itaconic acid. Therefore, product inhibition and potential influences resulting from three exemplary pretreatment methods were investigated, including high salt concentrations and residues from organic acid pretreatment. In addition, the ability of *U. maydis *to grow on pretreated hemicellulose from beech wood was studied.

## Results and discussion

### Reference cultivation of *Ustilago maydis *on glucose

A reference cultivation of *Ustilago maydis *MB215 was performed in Tabuchi medium with 120 g/L glucose and 1.6 g/L NH_4_Cl and cultivated in the Respiration Activity MOnitoring System (RAMOS) which allows the online monitoring of the respiration activity in shake flasks. The RAMOS monitors three important culture parameters: the oxygen transfer rate (OTR), the carbon dioxide transfer rate (CTR), and the respiratory quotient (RQ) [16]. The oxygen transfer rate is a common parameter often assessed in fermenters to characterize aerobic cultures. It includes all oxygen consuming metabolic activities. With the help of the OTR, complex phenomena such as diauxic growth, oxygen limitation or secondary substrate limitation can be detected [16]. By measuring the carbon dioxide transfer rate, the microbial carbon dioxide production is determined which indicates the microbial degradation of substrate. The respiratory quotient, provided by the CTR to OTR ratio, can reveal the current metabolic state of the microorganism, e.g. the type of substrate which is consumed or if overflow metabolites are produced. For more details, please refer to the paper of Anderlei et al. [16]. In addition, samples were drawn from parallel shake flasks to determine dry cell weight (DCW), cell number, and pH as well as the concentrations of ammonium, glucose, and itaconic acid.

**Figure 1:**
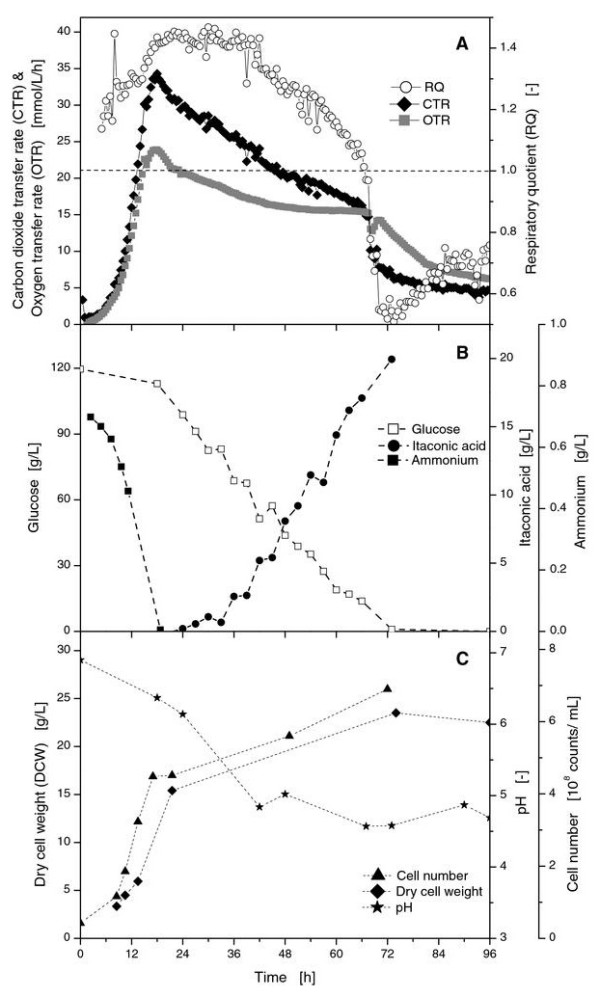
**Online measurements of OTR, CTR, and RQ for *U. maydis *MB215 reference culture in Tabuchi medium with 120 g/L glucose and 1.6 g/L NH_4_Cl (A)**. Glucose, itaconic acid, and ammonium concentrations from parallel shake flask experiments **(B)**. Cell number, dry cell weight, and pH value from parallel shake flask experiments **(C)**. Culture conditions: 250 mL RAMOS shake flasks, V_L _= 20 mL, n = 300 rpm, shaking diameter = 50 mm, T = 30°C.

Figure 1A shows the respiration activity of an *U. maydis *MB215 culture. In the first 16 hours, the CTR and OTR rose exponentially until a short decrease in the curve was visible. Afterwards, both transfer rate curves increased significantly slower while RQ increased from 1.1 to 1.3. After 18 hours, the maximum OTR and CTR values reached 24 mmol/L/h and 34 mmol/L/h, respectively. At the same time, the RQ increased from 1.3 during the exponential growth phase to 1.4 at the time point of maximum respiration activity which likely resulted from the production of reduced components and from the dissolution of the lime buffer. During the further cultivation, the CTR decreased almost linearly from 34 to 15 mmol/L/h after 70 hours. At that time, a sharp drop to a CTR of 7 mmol/L/h occurred. By contrast, the OTR-decrease slowed down until a plateau was maintained at 15.5 mmol/L/h. Therefore, the RQ slowly decreased from 1.4 to 1.2, indicating the production of less reduced components [16]. Finally, a strong decrease in the OTR was visible after 70 hours. At this time point, RQ also dropped from 1.2 to 0.6. The respiration remained on a low level but was above zero. To fully understand the development of the respiration activity, samples were drawn from parallel shake flasks.

As Figure 1B illustrates, two different nutrients mainly influenced the respiration activity. First, ammonium was depleted at the end of the exponential growth phase. Its depletion in the cultivation medium was shown to cause the characteristic break in the OTR curve in several experiments after ca. 16 hours. On the other hand, glucose was depleted after about 70 hours. Therefore, respiration activity decreased rapidly at this time point. After nitrogen was depleted, itaconic acid could be detected. Its concentration increased almost linearly to a final concentration of 20 g/L. Calculated over the whole cultivation time, this corresponded to an itaconic acid production rate of 0.27 g/L/h and a yield Y_P/S _of 0.17 g itaconic acid per g glucose. As already known for *U. maydis*, malic acid and succinic acid could be identified as fermentation by-products [12]. Although these results cannot compete with industrial *A*. *terreus *strains optimized over many years which reach final concentrations of more than 50 g/L, it should be noted here that our investigations were performed with a wild type strain under non-optimized conditions. However, the main focus of this study was to basically understand *U. maydis *and to determine how itaconic acid production was affected by different factors.

Parallel sampling also allowed the monitoring of pH development and biomass concentration (Figure 1C). After inoculation, the pH value of the cultivation medium was 6.8. During the first 36 hours, the pH-value decreased to 4.9 and stayed in the range between 4.8 and 5.0 for the remaining cultivation time. Apparently, the applied lime buffer concentration of 33 g/L guaranteed a sufficient and stable pH for the production of itaconic acid over the whole cultivation time. The production of itaconic acid did not seem to influence the pH value.

The dry cell weight (DCW) increased exponentially in the first 18 hours of the cultivation. Despite the nitrogen limitation at 16 hours, the DCW still increased from 15 to 23 g/L during the remaining cultivation time (Figure 1C). The cell counts of the samples were measured during cultivation with a Coulter Counter device. During the exponential growth phase, the respective cell number increased from 0.42 × 10^8 ^cells/mL after inoculation to 4.5 × 10^8 ^cells/mL at the point of ammonium depletion at 16 h (Figure 1C). In accordance to the DCW, the cell number still slightly increased after nitrogen limitation to 5.6 × 10^8 ^cells/mL.

Further experiments were performed to investigate the increase in the DCW as well as in the cell number in the phase of nitrogen limitation. As the RAMOS technology enables precise sampling at defined cultivation points, two samples were drawn from the nitrogen unlimited growth phase after 12 hours as well as from the nitrogen limited stationary phase after 28 hours. The *U. maydis *biomass of these samples was analysed regarding its elemental composition to determine carbon, hydrogen, oxygen, nitrogen, and sulphur contents (see Table 1). Compared to each other, both samples of *U. maydis *biomass significantly differed with regard to their elemental compositions. When the culture suffered from nitrogen limitation, the percentages of carbon and hydrogen rose whereas the oxygen and especially nitrogen contents drastically decreased. In other words, nitrogen-limited *U. maydis *cells contained 60% less nitrogen than that of unlimited cultures. Therefore, carbon-nitrogen (C/N) weight ratio increased from 5.9 to about 20. Consequently, the additional fungal biomass grown in the phase of nitrogen limitation was most likely generated through a "dilution" of nitrogen in the cells. This phenomenon allowed another reproduction cycle after ammonium was depleted from the media. Compared to elemental stoichiometric compositions of other species such as *S. cerevisiae *(C_1 _H_1.745 _O_0.673 _N_0.129_) [17], *U. maydis *showed a slightly higher content of hydrogen during its unlimited growth phase (C_1 _H_1.826 _O_0.5 _N_0.136_). Under nitrogen limited conditions, however, this ratio drastically changed to C_1 _H_1.925 _O_0.35 _N_0.039_. Anastassiadis [18] reported for *Candida oleophila *a similar pattern (C_1 _H_1.85 _O_0.33 _N_0.05_) for the production phase (idiophase), leading to a decrease in nitrogen content from 7.45% to 3.96% (w/w). Therefore, some microorganisms can compensate for limited nitrogen access to a certain degree by utilizing internal nitrogen pools. As the relative oxygen content of the fungal cells also decreased, the biomass contained more reduced and less oxygenized components which indicated an accumulation of hydrocarbons. This fact would explain the further increase in DCW at constant cell numbers and the high RQ of 1.4. In addition, the accumulation of hydrocarbons may also be a possible explanation for the shift towards larger cell sizes towards the end of the cultivation (data not shown). For *Aspergillus niger*, Kristiansen and Sinclair observed a similar correlation between the production of citric acid and the internal storage of carbon under nitrogen limitation [19]. Therefore, it can be assumed that nitrogen limitation not only induces the production of organic acids, but also induces an accumulation of hydrocarbons in *U. maydis*.

**Table 1:** Elemental composition of *Ustilago maydis *in exponential growth phase and under nitrogen-limited conditions

	Carbon	Hydrogen	Oxygen	Nitrogen	Sulphur	
	[weight %]	[weight %]	[weight %]	[weight %]	[weight %]	
Exponential phase	43.3 ± 0.2	6.59 ± 0.02	33.4 ± 0.1	7.35 ± 0.05	0.292 ± 0.012	

Under nitrogen imitation	57.9 ± 0.2	9.29 ± 0.07	27.0 ± 0.1	2.83 ± 0.05	< 0.2	

For the production of organic acids such as the desired itaconic acid, secondary substrate limitations, therefore, play a key role. Levison et al. reported about the production of itaconic acid for the basidiomycete *Pseudozyma antarctica *under nitrogen-limited conditions [20]. As *U. maydis *and *P. antarctica *are supposed to be close relatives, it was not surprising that the general patterns of itaconic acid production resembled each other. For the industrial production of itaconic acid with *A. terreus*, phosphate-limited conditions are applied [6,21]. To evaluate the influence of a phosphate limitation, the phosphate concentration was varied for *U. maydis *in another experiment. Hereby, KH_2_PO_4 _in concentrations below 0.25 g/L was shown to limit the respiration activity, but did not induce the production of itaconic acid. However, it did induce the production of numerous other organic acids (data not shown). In addition, phosphate limitation caused the cells most likely to accumulate even higher amounts of hydrocarbons which made the cells less dense than water. During centrifugation, these cells accumulated above the aqueous phase. Therefore, phosphate was excluded as a potential alternative for the secondary substrate limitation, at least in the wild type strain used in this work.

### Influence of nitrogen supply

**Figure 2:**
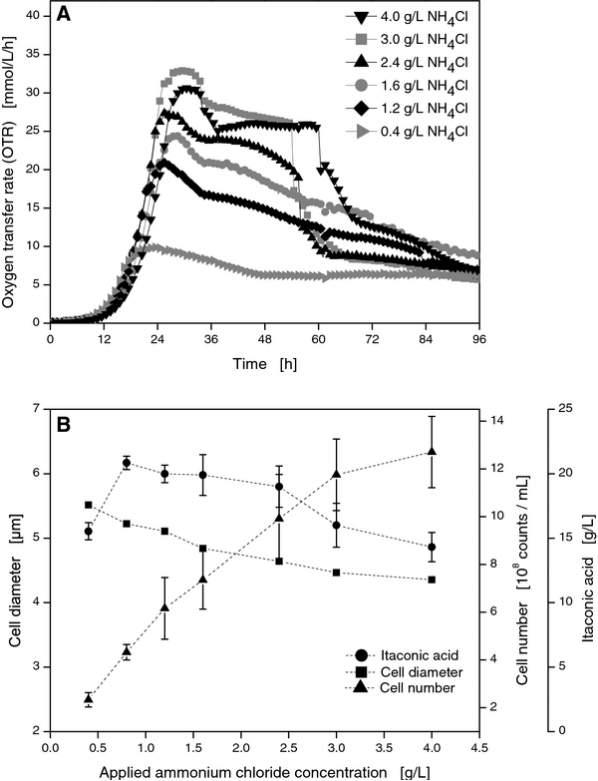
**Online measurement of OTR for *U. maydis *MB215 cultures in Tabuchi medium with 120 g/L glucose and varied ammonium chloride concentrations (A)**. Cell number, itaconic acid concentration, and cell diameter from parallel shake flask experiments as a function of the applied NH_4_Cl concentration **(B)**. Culture conditions: 250 mL RAMOS shake flasks, V_L _= 20 mL, n = 300 rpm, shaking diameter = 50 mm, T = 30°C.

As nitrogen is a crucial nutrient for culture growth, the influence of varied nitrogen concentrations was investigated. Ammonium chloride was applied at initial concentrations of 0.4 g/L, 1.2 g/L, 1.6 g/L, 2.4 g/L, 3.0 g/L, and 4.0 g/L in another set of RAMOS experiments as depicted in Figure 2A.

In the *U. maydis *cultivation with 0.4 g/L ammonium chloride, the culture suffered under a severe nitrogen limitation which already occurred after 16 hours. After 18 hours, the OTR reached its maximum at 10 mmol/L/h. When cultivation was terminated, still no signs of glucose depletion were visible in the respiration activity. For 1.2 g/L ammonium chloride, the OTR maximum was monitored at 20 mmol/L/h after 24 hours. As indicated by the reduced maximum OTR, lower ammonium concentrations resulted in less biomass formation. Therefore, the cultures needed more time to consume their substrate, resulting in increased cultivation times. With increasing ammonium concentrations, the OTR maximum increased from 25 mmol/L/h for the reference culture (1.6 g/L NH_4_Cl) via 28 mmol/L/h (2.0 g/L NH_4_Cl) up to 31 mmol/L/h (3.0 g/L NH_4_Cl). The increased OTR resulted from a longer growth phase in which more biomass was formed. Since the cultures with additional ammonium depleted the remaining glucose faster, the final drop in the OTR occurred earlier. For ammonium chloride concentrations exceeding 3.0 g/L, additional nitrogen did not further enhance the respiration activity, indicating a limitation of another secondary substrate instead of nitrogen. A further increase in ammonium chloride up to 4.0 g/L was found to slightly delay the cultivation of *U. maydis*. Most probably, high ammonium chloride concentrations above 4 g/L inhibited *U. maydis*.

*U. maydis *cell numbers increased in accordance to the nitrogen supply from 2 × 10^8 ^per mL for 0.4 g/L NH_4_Cl to 13 × 10^8 ^counts per mL for 4.0 g/L NH_4_Cl (Figure 2B). Nevertheless, a saturation was observed for higher concentrations than 3.0 g/L. This supports the occurrence of a new growth limitation from some other nutrient which was not further identified. The cell diameter was found to be slightly smaller at higher ammonium concentrations (Figure 2B). In particular, the cell diameter decreased from 5.5 μm for the cultivation with 0.4 g/L NH_4_Cl to 4.3 μm for the cultivation with 4.0 g/L NH_4_Cl.

The final itaconic acid concentration was only slightly affected by changes in nitrogen provided that nitrogen-limiting conditions were applied (Figure 2B). Nevertheless, a low nitrogen supply decreased the respiration activity and increased the cultivation time. For 0.4 g/L NH_4_Cl, glucose was not completely utilized after 96 hours. Additional nitrogen shortened the cultivation time, increased the cell number and, therefore, the dry cell weight of *U*. *maydis*. However, lower itaconic acid yields were achieved at high nitrogen concentrations. Hence, NH_4_Cl concentration was kept constant at 1.6 g/L for the following experiments.

In contrast to the model organism *Schizosaccharomyces pombe *[22], nitrogen limitation apparently increased the cell size of *U. maydis *(Figure 2B). Coulter counter data revealed that the earlier the nitrogen limitation occurred, the larger the cells grew. As already mentioned, this effect might be caused by an internal accumulation of hydrocarbon, but it might also be attributed to changes in the morphology. In case of *Candida oleophila*, not only an accumulation of glycerol and lipids was reported under nitrogen limited conditions, but also glycolipids may be stored in lipid bodies [23]. For *U. maydis*, the production of large amounts of glycolipids was shown for nitrogen limited cultures at low pH [24]. Nevertheless, the production of glycolipids was still observed in this study (data not shown), although the pH did not drift below 4.7 (Figure 1B). Accordingly, the production of glycolipids was very likely in part responsible for the unexpectedly high RQ above 1.2 (Figure 1A). For the strict stoichiometric production of itaconic acid from glucose, a RQ of 0.67 can be assumed. Ideally, 1 mol C_6_H_12_O_6 _and 1.5 mol O_2 _form 1 mol C_5_H_6_O_4_, 3 mol H_2_O, and 1 mol CO_2 _which would lead to a CTR/OTR ratio of 0.67. In addition, the RQ is also influenced by the gradual dissolution of the applied lime buffer which also released carbon dioxide. After glucose was depleted at the end of the cultivation, internal pools of carbohydrates may be used as substrate which would also correlate with the RQ below 1.0 after 72 h (Figure 1A).

To avoid the formation of hydrocarbons and to increase the production of itaconic acid, a genetically modified *U. maydis *strain should be applied. For *A. terreus*, the enzyme cis-aconitic decarboxylase (CAD) was shown to be responsible for the itaconic acid formation [25]. The main advantage of industrial strains seemed to be a higher expression level for CAD [25]. Once the synthesis pathway of itaconic acid for *U. maydis *is fully understood, an overexpression of the responsible enzymes is a promising way to increase yields. As alternative, the pathways for the synthesis of hydrocarbons such as glycolipids could be interrupted or decelerated.

### Cultivation under increased osmolarity in sea water

Through processing of biomass as feedstock for the fermentation, additional substances such as salts may enter the fermentation process. Vom Stein et al. have investigated a salt-assisted organic acid pretreatment [13,14]. This pretreatment is either able to hydrolyse hemicellulose or cellulose, depending on the applied conditions. Moreover, the pretreatment enhances subsequent process steps by breaking down the biomass structure. As an alternative, the enzymatic hydrolysis of cellulose in seawater was investigated [26], thereby saving fresh water resources. Consequently, potential influences of pretreatment methods applying high salt concentrations were addressed in this work.

Since salts such as sodium chloride were used as a supporting agent for the pretreatment of biomass, high salt loads could enter the fermentation process. Additional desalting steps might be unnecessary if *U. maydis *would prove to withstand increased osmolarity. The cultivation medium was shown to have an inherent osmolarity of ~1 osmol/L (with 120 g/L glucose) which can already be considered as high osmolarity [27]. To evaluate the influence of even higher osmolarities, defined amounts of sodium chloride were added to the cultivation medium after sterilization. Thereby, cultivations with different osmolarities from 1-3.5 osmol/L were monitored using the RAMOS device.

**Figure 3:**
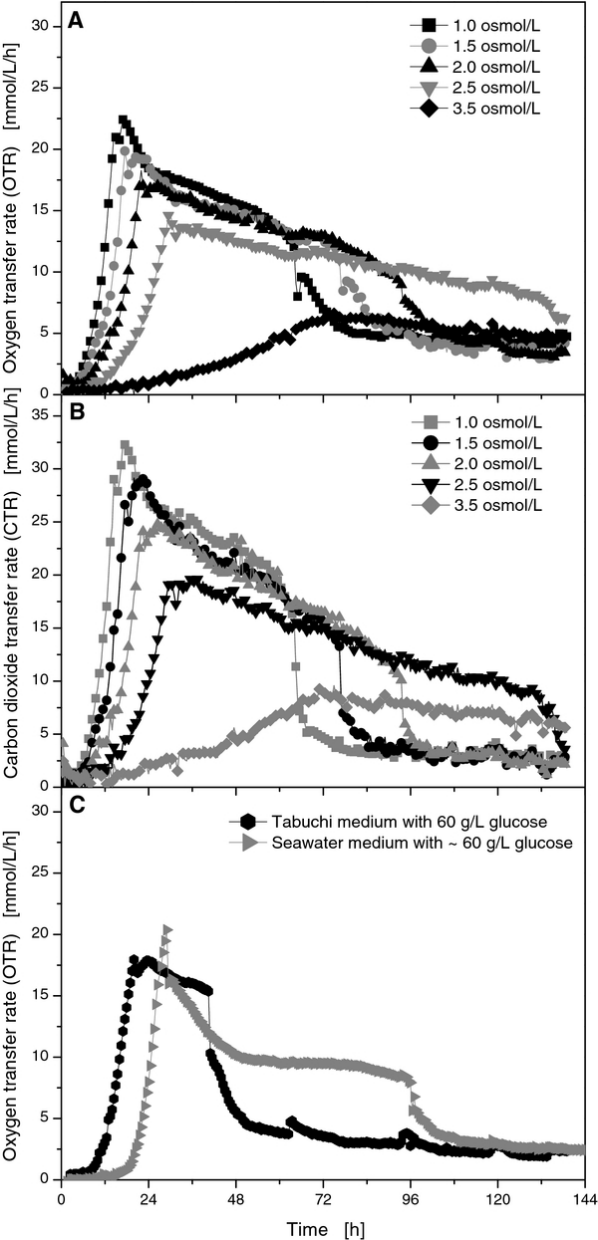
**Online measurement of OTR (A) and CTR (B) for *U. maydis *MB215 cultures in Tabuchi medium with 120 g/L glucose, 1.6 g/L NH_4_Cl, and varied osmolarities**. Online measurement of OTR for *U. maydis *MB215 cultures with ~60 g/L glucose and 1.6 g/L NH_4_Cl in Tabuchi medium and seawater medium (see materials & methods) **(C)**. Culture conditions: 250 mL RAMOS shake flasks, V_L _= 20 mL, n = 300 rpm, shaking diameter = 50 mm, T = 30°C.

As Figure 3A illustrates, the respiration activity clearly showed the influence of increased osmolarity. Compared to reference cultivation in Tabuchi medium without additional sodium chloride (~1 osmol/L), higher osmolarities delayed the exponential growth phase and decreased the maximum OTR. For the highest osmolarity 3.5 osmol/L (an addition of 73.15 g/L NaCl), the maximum OTR of 11.5 mmol/L/h was reached not until 42 hours. Hence, increased osmolarities hindered the growth of *U. maydis *and decreased its respiration activity in general. Under high osmotic conditions, microorganisms need adapt to the metabolic stress. According to the CTR, the cultivation time was significantly increased from 68 hours for the reference culture to 94 hours for an osmolarity of 2.0 osmol/L and more than 144 hours for an osmolarity of 3.5 osmol/L (Figure 3B). Nevertheless, *U. maydis *proved itself as a robust, osmo-tolerant organism, withstanding at least 73.15 g/L sodium chloride which represents twice the salt concentration of seawater (ca. 35 g/L).

Based on these results, cultivations with real seawater were performed. Thus, 100 g/L Sigmacell cellulose was depolymerized with Accellerase (Genencor, Leiden) as described in the material and methods part. HPLC measurements showed an approximate concentration of 60 g/L glucose in the seawater preparation. All other nutrients of the Tabuchi medium except glucose were added to the seawater to generate a suitable seawater medium. For comparison, experiments with Tabuchi medium and 60 g/L glucose were monitored in the RAMOS device (Figure 3C). By applying only 60 g/L glucose instead of 120 g/L glucose to the Tabuchi medium, the OTR increased after a lag phase of 11 hours to a maximum of 18 mmol/L/h. As expected for such a reduced glucose concentration, glucose was already exhausted after 46 h. During this time period, a maximum of 3.5 g/L itaconic acid was produced (results not shown). This concentration corresponded well with the itaconic acid production in the reference cultivation when 60 g/L glucose was consumed (Figure 1B). By cultivating *U*. *maydis *in pure seawater medium, a long lag phase 20 hours was determined. The OTR maximum was observed at 20.4 mmol/L/h. As indicated by the increased maximum OTR, the seawater medium contained slightly more ammonium than the normal medium resulting in a final ammonium chloride concentration of ~1.81 g/L. After 96 h, a final concentration of 6.4 g/L itaconic acid was detected. Unexpectedly, seawater seemed to slightly increase the overall production of itaconic acid. For *Candida oleophila*, Anastassiadis et al. reported an increased production of citric acid under osmotic shock conditions [18]. Since the exact synthesis pathway of itaconic acid is not yet fully understood for *U. maydis*, it can only be assumed that the secretion of itaconic acid might be accelerated under increased osmolarity. At the end of the experiment, the respective osmolarities were determined at 0.131 osmol/L for the Tabuchi medium and 1.539 osmol/L for the seawater medium.

In general, cultivation under increased osmolarity was possible. High amounts of salts could enter the process not only by seawater, but also by other methods such as acid pretreatment. For the fermentation process itself, high substrate concentrations as well as high buffer concentrations are known to cause osmotic stress. For bioreactors, the corrosive nature of saltwater also has to be considered. Nevertheless, enormous amounts of fresh water could be saved if seawater was used for fermentation media. In 2011, Lin et al. presented the production of succinic acid in synthetic sea water which was shown to act also as mineral supplement for the microorganism [28]. In summary, advantages and disadvantages of high salt concentrations have to be carefully balanced. However, utilizing an osmo-tolerant organism also mitigates other problems such as high initial substrate concentrations.

### Product inhibition by itaconic acid

Besides high salts loads, residues of organic acids from pretreatment supposedly influence *U*. *maydis*. Organic acids in the medium may not only inhibit growth, but production of itaconic acid may also be influenced. In fact, itaconic acid itself may affect its production. In process development, product inhibition is generally an important factor as it directly affects the obtainable product concentrations. Therefore, this obstacle is often targeted by research groups via genetic engineering [29]. For *A. terreus*, a screening for mutants with higher resistance to itaconic acid led to increased yields [30]. Although only moderate final concentrations for itaconic acid could be achieved with the applied *U. maydis *wild type strain, it was essential to determine a critical product concentration in this current study. Consequently, inhibitions of growth and of product formation were both investigated under increased itaconic acid concentrations. Different amounts of itaconic acid were added to the Tabuchi medium after autoclaving, but before inoculation. To avoid pH influences, the itaconic acid solutions were adjusted to the initial pH-value of the cultivation medium (6.8). As the addition of itaconic acid also increased the osmolarity, 53.9 g/L sodium chloride was applied to one flask to mimic the osmotic effect of 80 g/L itaconic acid. Before HPLC analysis, the pellets were washed several times to include eventually precipitated itaconic acid in the HPLC measurements.

**Figure 4:**
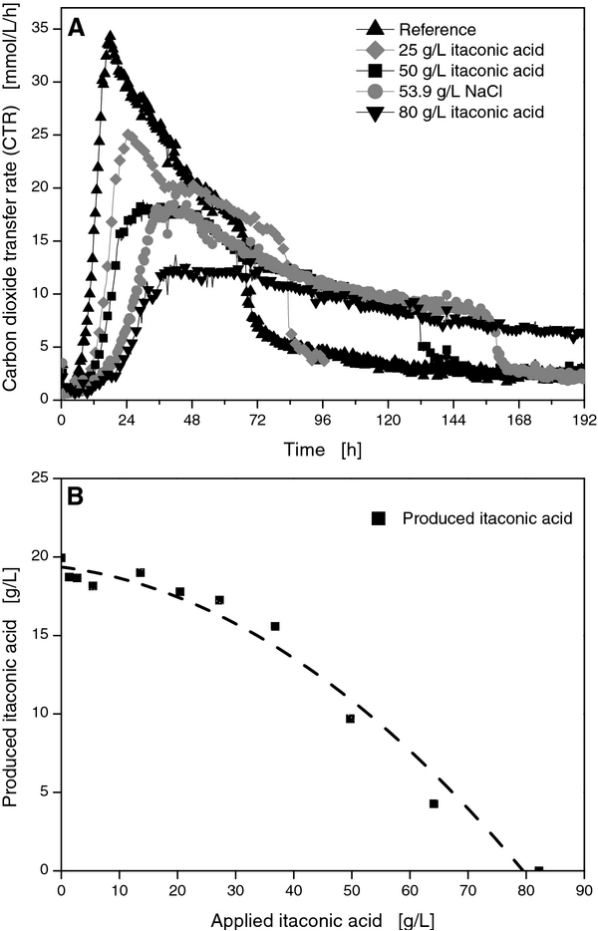
**Online measurement of OTR for *U. maydis *MB215 cultures in Tabuchi medium with 120 g/L glucose, 1.6 g/L NH_4_Cl, and varied initial itaconic acid concentrations**. To mimic the osmolarity of 80 g/L itaconic acid, 53.9 g/L NaCl was added to one culture **(A)**. Additional itaconic acid production as a function of the initial itaconic acid concentration **(B)**. Culture conditions: 250 mL RAMOS shake flasks, V_L _= 20 mL, n = 300 rpm, shaking diameter = 50 mm, T = 30°C.

The CTR depicted in Figure 4A showed strong influences of itaconic acid. High initial itaconic acid concentrations of 25 g/L and 50 g/L increased the cultivation time to 84 hours and 132 hours and reduced the maximum CTR to 25 mmol/L/h and 18 mmol/L/h, respectively. Although *U. maydis *was able to grow under the highest soluble concentration of itaconic acid (80 g/L), the maximum CTR decreased from 34 mmol/L/h to 12 mmol/L/h for 80 g/L itaconic acid, prolonging cultivation to more than 192 hours. To mimic the osmolarity of the highest itaconic acid concentration, 53.9 g/L sodium chloride was added. Nevertheless, the respective itaconic acid concentration of 80 g/L showed more pronounced effects on respiration activity than that of sodium chloride.

To evaluate product inhibition, the additionally produced amounts of itaconic acid were compared to the supplemented initial concentration (Figure 4B). For itaconic acid concentrations exceeding 25 g/L, an increasing product inhibition was detected leading to decreased yields and lower final concentrations of the actual product. At a maximum applied concentration of 80 g/L itaconic acid, the production was completely inhibited. Consequently, high itaconic acid concentrations should be either avoided by *in-situ *product removal or a more resistant strain has to be developed. Welter reported a strong inhibition of *A. terreus *already at comparatively low concentrations of 10 g/L itaconic acid [31]. Nevertheless, final product concentrations over 80 g/L have been achieved after strain improvement [30].

### Fermentation on pretreated substrates

**Figure 5:**
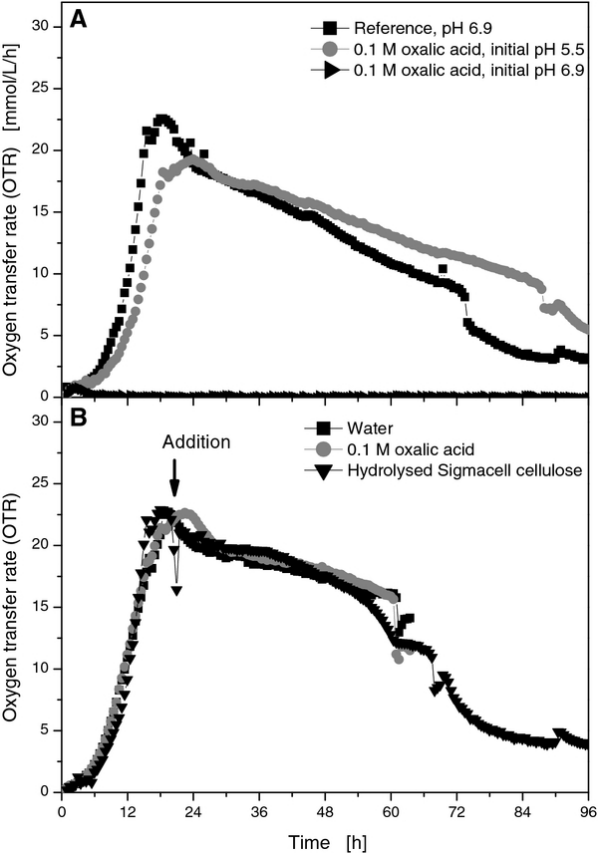
**Online measurement of OTR for *U. maydis *MB215 cultures in Tabuchi medium with 120 g/L glucose, 1.6 g/L NH_4_Cl**. Reference culture with initial pH 6.9, cultures with 0.1 M oxalic acid at an initial pH of 5.5 (shift to 6.7) and with an initial pH of 6.9 (shift to 8.9) (A). Addition of 5 mL water, 0.1 M oxalic acid, and hydrolysed Sigmacell cellulose after 18 hours **(B)**. Culture conditions: 250 mL RAMOS shake flasks, V_L _= 20 mL (25 mL after addition), n = 300 rpm, shaking diameter = 50 mm, T = 30°C.

For the salt-assisted organic-acid catalysis [13], various organic acids have been evaluated as potential catalysts. Thereby, oxalic acid was found to be one of the most promising dicarbonic acids. However, carboxylic acids can have toxic effects on microorganisms [32]. Even though oxalic acid can optionally be precipitated after the pretreatment and removed, its influence on *U. maydis *was monitored in the RAMOS device. Therefore, different amounts of oxalic acid were applied to the cultivation medium before inoculation and compared to the reference cultivation. If added before inoculation, even comparatively small amounts of oxalic acid of around 0.1 M completely inhibited the respiration activity and, therefore, the growth of *U. maydis *(Figure 5A). This massive impact was precisely investigated and could be attributed to a pH increase. Although the oxalic acid solution was adjusted to pH 6.9 before addition, the pH of the medium slowly increased over several hours from 6.9 to 8.5. Apparently, this increasing alkalinity was caused by the gradual precipitation of calcium oxalate in the presence of the lime buffer. To compensate for this effect, the pH-value of the oxalic acid solution was adjusted before the RAMOS experiment (Figure 5A). In this case, a comparatively small impact could be registered for an addition of 0.1 M oxalic acid. The OTR maximum was shifted to 19.1 mmol/L/h after 29 hours. Consequently, the cultivation time increased to 90 h. HPLC analysis revealed a final concentration of 16 g/L itaconic acid for this culture whereas the reference culture reached a final concentration 20 g/L.

To monitor the direct influence of oxalate on *U. maydis*, 5 mL of a 0.1 M oxalic acid solution was added after nitrogen limitation occurred, and this addition was compared to an addition of 5 mL water (Figure 5B). Moreover, 10 g/L pretreated cellulose from the salt-assisted organic-acid catalysis was added to another culture which equals 11 g/L glucose. Thereby, also 0.1 M oxalic acid was applied. If added after the nitrogen limitation at 18 hours, oxalic acid had only a minimal influence on the respiration activity. The OTR maximum was slightly delayed by four hours, but it also reached 22.5 mmol/L/h. Furthermore, no influence on the production of itaconic acid was detected, since the final itaconic acid concentrations were comparable to that of the reference culture that was spiked with water (data not shown). As the addition of 5 mL water or oxalic acid solution to 20 mL cultivation medium decreased the glucose concentration due to dilution, both cultures exhausted their glucose already after 64 hours. After the addition of salt-assisted organic-acid catalysed cellulose, the respiration activity curve showed a short break at an OTR of 15 mmol/L/h. Nevertheless, the OTR recovered to its former level of around 20 mmol/L/h after 1 h.

Since the addition of oxalic acid would inevitably interfere with the lime buffer, it was difficult to evaluate a critical concentration of oxalic acid. At least for the shake flask system, the results that indicated the most harmful effect of oxalic acid seemed to be its alteration of the pH-value. Although this effect has to be considered and prevented for small scale cultivations, larger fermentations in stirred-tank bioreactors are not affected due to their pH regulation and the absence of lime. Therefore, low concentrations of oxalic acid might be tolerable if they sufficiently enhance the pretreatment of the biomass. In principle, the salt-assisted organic-acid catalysis could replace an enzymatic hydrolysis of cellulose. Nevertheless, the experiments indicated that subsequent reactions and formations of humic acids during the pretreatment drastically reduced the available glucose. Most probably, these side reactions were caused by the combination of high temperatures and long reaction time. As it should be noted that the salt-assisted organic-acid catalysis method is a non-optimized proof of principle at the moment, this pretreatment might be improved by integrated solutions such as an *in-situ *removal of the glucose. In general, cellulose should be depolymerized under mild conditions in the future, either chemically or enzymatically.

In general, *U. maydis *proved to be a robust organism to cope with the various influences originating from biomass pretreatment. To demonstrate the general feasibility of real biomass as substrate for *U. maydis*, the hemicellulose fraction from the selective organic acid-catalysed depolymerisation of hemicellulose was chosen for a preliminary investigation [13]. Not only has this process shown to generate decent amounts of fermentable xylose, also no side-reactions to unwanted by-products such as humic acids were observed. As it is known that *U. maydis *can utilize xylose, a set of RAMOS experiments was performed to monitor the respiration activity of *U. maydis *on commercial D-xylose and glucose-xylose mixes. In other words, 90 g/L glucose was mixed with 30 g/L D-xylose for one culture while the other cultivation was performed with a mixture of 60 g/L of each sugar.

**Figure 6:**
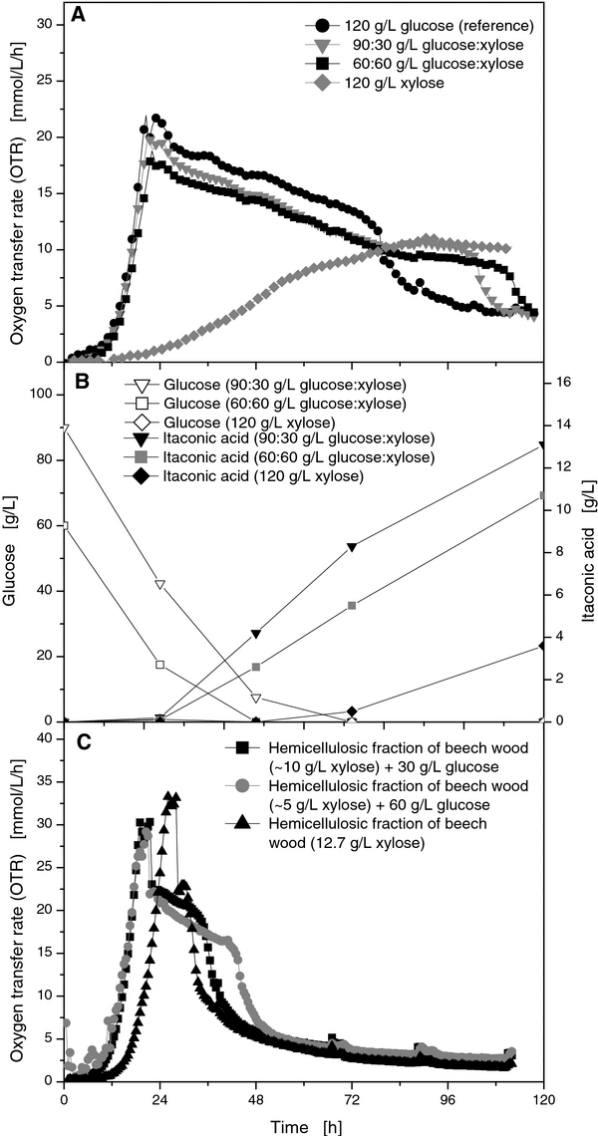
**Online measurement of OTR for *U. maydis *MB215 cultures in Tabuchi medium with 1.6 g/L NH_4_Cl and different ratios of glucose and xylose (A), respectively different ratios of the hemicellulosic fraction of beech wood and glucose (B)**. Culture conditions: 250 mL RAMOS shake flasks, V_L _= 20 mL (25 mL after addition), n = 300 rpm, shaking diameter = 50 mm, T = 30°C.

When *U. maydis *was solely cultivated on xylose, it grew considerably slower compared to a cultivation on glucose (Figure 6A). After 90 hours of an almost linear increase in the OTR curve, the maximum respiration activity was found at an OTR of 11 mmol/L/h. Further experiments with both xylose and glucose showed a much higher respiration activity, resembling that of the reference culture growing exclusively on glucose. If glucose was present in the media, the initial lag and exponential growth phase were almost identical for the aforementioned cultures. Therefore, *U. maydis *prefers glucose over xylose. With an increasing concentration of xylose, the maximum OTR slightly decreased from 22 mmol/L/h to 20 mmol/L/h and 19 mmol/L/h. Subsequently, all cultures showed a gradual decrease in respiration activity with no clear indication when the change from glucose to xylose occurred.

By HPLC analysis, glucose was shown to be completely consumed in the first ~40 hours for an initial glucose concentration of 60 g/L (Figure 6B) and in the first ~65 hours for initial glucose concentration of 90 g/L. Therefore, the remaining respiration activity was generated by *U. maydis *consuming the xylose. In accordance to their lower respiration activities, the cultures growing on mixed substrates depleted their C-sources at a later point (Figure 6A). Hence, *U. maydis *could utilize xylose, but showed a very slow growth on this substrate. In the presence of additional glucose, the fungus could grow on its preferred substrate and exhibited no significantly diminished respiration activity during the subsequent consumption of xylose. The production of itaconic acid continued after glucose was depleted (Figure 6B), although lower yields were accomplished compared to the reference cultivation on glucose. The culture with 90 g/L glucose and 30 g/L xylose yielded 13.1 g/L itaconic acid, whereas the culture with 60 g/L glucose and 60 g/L xylose had a final concentration of 10.7 g/L. On sole xylose, the slow growth also prevented the complete utilization of the xylose which is why a low final concentration of 3.6 g/L itaconic acid was achieved. Therefore, it can be assumed that xylose can act as substrate for the production of itaconic acid, but is less preferable than glucose.

The hydrolysed hemicellulose fraction of beech wood was applied as substrate in a RAMOS experiment. The xylose concentration was determined to be ~13 g/L by HPLC (data not shown). Small amounts of glucose were also present in this fraction (< 2 g/L). As illustrated in Figure 6C, exponential growth phase started after a lag phase of 10 hours if the hydrolysed hemicellulose was applied as sole carbon source. After 32 h, respiration activity decreased again. As was indicated by the increase in the maximum OTR of 32.3 mmol/L/h, the hydrolysed hemicellulose fraction contained additional nitrogen (0.2 g/L nitrate). Due to the low sugar concentrations and a consequential very short production phase after the OTR maximum, the final itaconic acid concentration was low at 0.36 g/L for the hydrolysed hemicellulose as sole C-source. In some experiments, additional pure glucose was supplemented to mimic a potential complete utilization of the cellulose fraction. By utilizing both the cellulosic pulp fraction and the hemicellulose fraction for fermentation, an approximate xylose-glucose-ratio of 1:3 would be expected for beech wood. When 10 g/L hydrolysed hemicellulose was combined with 30 g/L additional glucose, lag phase was reduced to six hours, followed by an exponential growth phase up to an OTR of 30.2 mmol/L/h (Figure 6C). At the end of the cultivation after 36 h, an itaconic acid concentration of 4.1 g/L was found. For the mixture of 5 g/L hydrolysed hemicellulose and 60 g/L of glucose, a very similar OTR curve was monitored but the sharp decrease in the OTR was delayed until 44 h. This mixture yielded 8.5 g/L itaconic acid.

In summary, the combined utilization of the cellulosic fraction and of the hemicellulosic fraction of beech wood is reasonable for a decent production of itaconic acid. Moreover, the low initial sugar concentrations have to be increased for a suitable process to elongate the production phase. Nevertheless, the general feasibility of fermentation was proven for the hydrolysed hemicellulose. Therefore, the selective organic acid-catalysed depolymerisation was exemplarily shown to yield a fermentable hemicellulose fraction of beech wood.

Until now, industrial processes for the production of itaconic acid with *A. terreus *have relied on the fermentation of hydrolysed starch or molasses [7]. Several studies presented itaconic acid fermentations from partially hydrolysed starch [33,34]. Petruccioli et al. compared different starchy materials and pointed out the relevance of a sufficient hydrolysis [35]. In contrast, processes with lignocellulose as raw material for the itaconic production are rarely described although conversions of xylose to itaconic acid are described for *A. terreus*. As an exception, Kobayashi already proposed a process using xylose from soft wood in 1978 [15]. In our study, the direct production of itaconic acid from the hemicellulosic fraction of selectively fractionated hardwood was successful, even though the applied pretreatment methods are not yet tailor-made for biotechnological processes.

### *Ustilago maydis *as production organism for itaconic acid

*U. maydis *was shown to be a promising producer of itaconic acid. Although the *U. maydis *wild type strain MB215 only produced relatively low final product concentrations of itaconic acid as compared to existing industrial processes with *A. terreus*, genetic modifications might considerably improve the itaconic acid production in the future. In 2002, Reddy and Singh showed the improvement of an *A. terreus *wild type strain by different mutagenic treatments [36]. Thereby, the final itaconic acid concentration of 30 g/L obtained from 120 g/L hydrolysed starch was increased to 48 g/L. For *U. maydis*, much better yields will be obtained if the undesired by-products such as malic acid and glycolipids are eliminated.

Moreover, the economic viability of a process not only depends on yields, but also but also on space-time-yield and on the chosen substrate. In the future, the increasing demand on cheap substrates and the concurrence for existing substrates will likely boost the relevance of other attributes. Rumbold et al. stated in their study [37] that in addition to the basic performance of a production organism in controlled fermentations, the use of carbon source and its resistance to inhibitors are also important benchmarks for a production organism. Unlike *A. terreus *which produces itaconic acid at pH values below 3, *U. maydis *produced itaconic acid at moderate pH-values of between 4.5 and 6. For a potential application of a SSF process (Simultaneous Saccharification and Fermentation), process conditions will have to match both the microorganism as well as the cellulases. Therefore, the fermentation conditions will have to match the pH-optima of cellulases around 4.8. This prerequisite applies more for *U. maydis *than for *A. terreus *as commonly available cellulases would not perform well under pH values below 3 [38]. In addition, the advantage of the yeast-like, single cell growth of *U. maydis *will facilitate a scale-up in larger stirred tank bioreactors. For *A. terreus*, air lift bioreactors were shown to yield better fermentation results than stirred tank reactors [39], most likely due the sensibility of *A. terreus *mycelia to hydromechanical stress [40]. In contrast, interaction between cellulases and their substrates requires complete suspension of the cellulose and, therefore, high power input [41].

## Conclusions

*U. maydis *combines important advantages of yeasts - a non-filamentous growth and resistance to hydromechanical stress - with the advantages of filamentous fungi - an inherent utilization of xylose and robustness against impurities from crude biomass feedstock. However, applying beech wood or other plant materials can interfere with the required nitrogen limitation. Consequently, composition of the applied material has to be evaluated when "pure" substrates such as glucose are replaced by complex sugar containing raw materials. Although *U. maydis *has proven itself to be insusceptible to possible impurities from pretreatment, it has been shown that pretreatment can significantly influence the subsequent fermentation process. In this study, online measurements with the RAMOS device have clearly demonstrated that chemical compounds from biomass pretreatment can inhibit microbial growth and the production of the desired product. Consequently, it is important to consider that a pretreatment method is only successful, if it does not inhibit the subsequent fermentation. Ultimately, mild pretreatment conditions and robust microbial producers such as *Ustilago maydis *will provide a good approach for a successful process development.

## Methods

### Microorganism

All experiments were performed with *Ustilago maydis *MB215 which was kindly provided by Michael Bölker, Philipps-Universität Marburg, Germany. This wild type strain, isolated in Northern Germany, is available at the German Collection of Microorganisms and Cell Cultures (DSMZ) under accession number DSM17144 (MB215) [24].

### Cultivation and media

All cultures were performed in 250 mL shake flasks with a filling volume of 20 mL at 30°C and were shaken at a shaking frequency (n) of 300 rpm with a shaking diameter (d_0_) of 50 mm. For both precultures and main cultures, Tabuchi medium [12] was used which consists of 120 g/L glucose, 1.6 g/L NH_4_Cl, 0.5 g/L KH_2_PO_4_, 0.2 g/L MgSO_4_*7H_2_O, 1 g/L yeast extract (Roth, Karlsruhe, Germany) and 10 mg/L FeSO_4_*7H_2_O. The medium was buffered with 33 g/L lime (CaCO_3_) (Roth, Karlsruhe, Germany). The iron sulphate and lime buffer were sterilized separately and added after autoclaving. Precultures were inoculated from cryo cultures, whereas main cultures were inoculated with 200 μL from a three day old preculture.

### Respiration activity monitoring system

To monitor the respiration activity, an in-house manufactured Respiration Activity MOnitoring System (RAMOS) device was used. This device can measure the oxygen transfer rate (OTR) and the carbon dioxide transfer (CTR) [16,42]. Therefore, the respiratory quotient (RQ) can also be calculated which gives additional information about the metabolic status of a culture. The RAMOS is capable of monitoring noninvasively up to eight parallel shake flasks. A commercial version of the RAMOS device can be obtained by HiTec Zang GmbH, Herzogenrath, Germany or Kühner AG, Birsfelden, Switzerland.

### Sample analytics

Samples were drawn from parallel cotton plug-sealed shake flasks since direct sampling would interfere with the RAMOS measurement. Of each flask, 2 mL culture broth was taken and centrifuged at 10.000 rpm for 5 minutes. The respective supernatant was used for other assays, whereas the pellet was resuspended in 0.5 M acetic acid for 10 minutes to remove remaining lime particles. It was then centrifuged again and dried for at least 48 hours in a 60°C cabinet. Subsequently, the pellet was weighed to determine dry cell weight (DCW). As evaporation influenced the final concentration of itaconic acid and the DCW, all concentrations were recalculated according to the remaining filling volume in the shake flasks.

The pH-value of the supernatant was measured with a CyberScan pH 510 (Eutech, Nijkerk, The Netherlands). To quantify glucose, itaconic acid, and other organic compounds, the supernatants were analysed with High Performance Liquid Chromatography (HPLC). The HPLC analysis was performed with a Dionex HPLC (Dionex, Sunnyvale, USA) with an Organic Acid-Resin 250 × 8 mm (CS-Chromatographie, Langerwehe, Germany) and a Skodex RI-71 detector. As solvent, 5 mM H_2_SO_4_, with a flow rate of 0.6 mL/min and a temperature of 60°C, was used. Ammonium concentrations were determined in a Spectroquant Nova 60 photometer (Merck, Darmstadt, Germany) using the photometric Ammonium Cell Test 114559 (Merck, Darmstadt, Germany).

The *U. maydis *biomass of some samples was analysed regarding its elemental composition in Jülich Research Center, Germany. Carbon, hydrogen, oxygen, nitrogen, and sulphur contents were hereby determined.

### Coulter counter

A Multisizer™ 4 COULTER COUNTER^® ^from Beckman Coulter, Krefeld, Germany, was utilized for cell counting. This device can count cells and simultaneously detect their size. An aperture with a diameter of 100 μm which can detect particles between 0.6 and 60 μm was applied. For each measurement, 20 μL culture broth was diluted in 200 mL Beckman Coulter electrolyte. Of this solution, 1 mL was drawn through the aperture and examined for particles. Particle-size distributions were analysed with the accompanying Multisizer 4 software. Although undissolved lime particles were present in the media, the *U. maydis *cells already outnumbered these lime particles at the time point of inoculation. Moreover, the size of most lime particles ranged between 15 and 30 μm diameter which clearly exceeded the size of *U. maydis *cells of 3-10 μm diameter. Therefore, all measured particles having a size of 3-10 μm were taken to be fungal cells.

### Osmolarity (Osmolality)

Osmolality was measured using an Osmomat 030 (Gonotec, Berlin, Germany) device. After a 2-point calibration, the supernatant was analysed via cryoscopy according to the manufacturers' protocol. As the osmolality is defined in osmol/kg and the density of the cultivation medium almost equals 1.0, all the data here are given as osmolarity in osmol/L.

### Pretreatment

To investigate how the different pretreatment methods affects the cultivation of *U. maydis*, three different pretreated biomass fractions were applied as substrates: 1) Sigmacell cellulose hydrolysed enzymatically in seawater, 2) Sigmacell cellulose hydrolysed via salt-assisted organic-acid catalysis, and 3) monomeric hemicellulose from fractionated beech wood.

To hydrolyse cellulose enzymatically in seawater, 100 g/L amorphous cellulose (type 101, Sigmacell) was treated with 1 vol% Accellerase 1500 (Genencor, a Danisco Division, Palo Alto, CA., USA) [26]. The reaction was buffered at pH 4.5 with a 0.1 M citrate buffer and performed at 50°C for seven days. Afterwards, the resulting seawater mixture was used by itself instead of deionized water to prepare the Tabuchi medium, generating the so-called seawater medium. As the cellulose was not completely hydrolysed, this medium was shown by HPLC measurements to contain 60 ± 5 g/L glucose [26]. For better comparability, the glucose concentration of the Tabuchi medium was also reduced to 60 g/L for this experiment.

To perform the salt-assisted organic-acid catalysed hydrolysis of cellulose [14], 50 g/L amorphous cellulose (type 101, Sigmacell) was hydrolysed with 0.1 M oxalic acid and 50 g/L sodium chloride at 125°C at 10 bar for 7 days.

According to vom Stein et al., the liquefied hemicellulosic fraction of beech wood contained 18 ± 0.3 wt(%) xylose and 2.0 ± 0.2 wt(%) glucose [14]. To generate the hemicellulose fraction by selective organic acid-catalysed depolymerisation [13], 50 g/L beech wood was fractionated with 0.1 M oxalic acid at 125°C and 10 bar CO_2 _for six hours. As solvent, a 1:1 mixture of water and 2-MTHF was applied. After six hours, the organic 2-MTHF phase containing the lignin was separated by decantation. The aqueous phase was filtered to remove the solid cellulose pulp. Oxalic acid was precipitated by adding CaCl_2_. Subsequently, the water from the aqueous phase was completely evaporated and the remaining hemicellulose sugars were used as substrate for the preparation of the Tabuchi medium. For the cultivation with sole beech wood, a xylose concentration of 12.8 g/L was determined.

## Abbreviations

*U. maydis*: *Ustilago maydis; *RAMOS: Respiration activity monitoring system; OTR: Oxygen transfer rate; CTR: Carbon dioxide transfer rate; RQ: Respiratory quotient; MTHF: Methyltetrahydrofuran; DCW: Dry cell weight.

## Competing interests

The authors declare that they have no competing interests.

## Authors' contributions

TK designed this study, participated in the RAMOS experiments and drafted the manuscript. SM carried out the RAMOS experiments. GJ participated in design of study and the data interpretation. PG carried out the three different pretreatment methods which were designed by PD. JB supervised the study and assisted in drafting the manuscript. All authors read and approved the final manuscript.
